# Enhancing Ecological Validity: Virtual Reality Assessment of Executive Functioning in Children and Adolescents with ADHD

**DOI:** 10.3390/children11080986

**Published:** 2024-08-14

**Authors:** Dulce Romero-Ayuso, Antonio del Pino-González, Antonio Torres-Jiménez, Jorge Juan-González, Francisco Javier Celdrán, María Constanza Franchella, Nuria Ortega-López, José Matías Triviño-Juárez, Ana Garach-Gómez, Luisa Arrabal-Fernández, Inmaculada Medina-Martínez, Pascual González

**Affiliations:** 1 Department of Physical Therapy, Occupational Therapy Division, University of Granada, 18071 Granada, Spain; antoniodelpino@correo.ugr.es (A.d.P.-G.); nonotorres@correo.ugr.es (A.T.-J.); e.franchella@go.ugr.es (M.C.F.); nortega@correo.ugr.es (N.O.-L.); 2 Instituto de Investigación Biosanitaria de Granada, Ibs Granada, 18012 Granada, Spain; 3 Brain, Mind and Behaviour Research Center (CIMCYC), University of Granada, 18011 Granada, Spain; 4 Department of Computing Systems, University of Castilla-La Mancha, 02006 Albacete, Spain; jorge.juan@uclm.es (J.J.-G.); franciscoj.celdran@uclm.es (F.J.C.); pascual.gonzalez@uclm.es (P.G.); 5 Department of Radiology and Physical Medicine, Faculty of Medicine, University of Granada, 18016 Granada, Spain; jmtjuarez@ugr.es; 6 Centro de Salud Zaidín Sur, Servicio Andaluz de Salud, 18007 Granada, Spain; ana.garach.sspa@juntadeandalucia.es; 7 Servicio de Neuropediatría, Hospital Universitario Virgen de las Nieves, 18014 Granada, Spain; luisam.arrabal.sspa@juntadeandalucia.es (L.A.-F.); inmaculada.medina.sspa@juntadeandalucia.es (I.M.-M.)

**Keywords:** assessment, executive functions, ADHD, virtual reality, activities of daily living

## Abstract

Background: SmartAction-VR uses virtual reality to simulate daily life tasks and assess cognitive performance based on the multi-errand paradigm. This study explored whether this new task could provide insights into the executive functioning of children and adolescents with ADHD in their everyday activities. Methods: A cross-sectional study was conducted between November 2021 and December 2022. It consisted of one session and was divided into two parts (cognitive tests; and SmartAction-VR). The sample comprised 76 children and adolescents with a median age (IQR) of 13 (11–14) years and an age range of 9–17 years. Of these participants, 60.50% (*n* = 46) were males. Out of this sample, 40 participants were in the ADHD group and 36 were in the neurotypical group. The following instruments were used: Waisman Activities of Daily Living Scale, Assessment of Sensory Processing and Executive Functioning, Pediatric Simulator Disease Questionnaire, Digit span subtest, Stroop test, NEPSY-II Subtest of Auditory Attention and Cognitive Flexibility, Trail Making Test, Zoo Map Test, and SmartAction-VR. Results: The ADHD group demonstrated lower accuracy (U = 406, *p* = 0.010), higher values for total errors (U = 292, *p* = 0.001), more commissions (U = 417, *p* = 0.003), new actions (U = 470, *p* = 0.014), and forgetting actions (U = 406, *p* = 0.010), as well as fewer perseverations compared to the neurotypical group (U = 540.5, *p* = 0.029). Additionally, participants who forgot more actions were found to have lower independence in daily life (r = −0.281, *p* = 0.024). Conclusions: The correlations between the results of SmartAction-VR and activities of daily living, as well as cognitive tests, suggest that this new task could be useful for evaluating executive functioning in daily life.

## 1. Introduction

Attention deficit hyperactivity disorder (ADHD) is a neuropsychiatric condition of considerable interest among health and education professionals because of the pervasive behavioral impairments it causes across various domains of daily life [1]. These difficulties can persistently and severely affect behavior, with a prevalence of approximately 5.9% among children and adolescents [2]. Early detection, facilitated by effective assessments of daily life impacts, can mitigate many consequences of inadequate interventions for children with ADHD. 

Traditional assessment methods for ADHD, which have been used since the 1980s, have highlighted the differences in executive functions (EFs) in people with ADHD compared with their neurotypical peers. These evaluations measure cognitive processes such as working memory, inhibitory control, cognitive flexibility, planning, action monitoring, and problem-solving [3]. However, their accuracy in assessing functional deficits in ADHD may be limited. Typically conducted in clinical or laboratory settings, these tests often evaluate specific skills in isolation, potentially overlooking interactions between different EFs and their relevance to daily activities [4,5]. Moreover, laboratory settings may influence a child’s behavior, increasing anxiety and affecting task performance, thus reducing predictive validity [6].

There is an increasing demand for assessments that identify ADHD-related difficulties in real-world settings and enable early ecologically valid predictions. The concept of ecological validity encompasses the representativeness of cognitive demands as they occur in the real world, emphasizing complexity and the inclusion of naturalistic tasks [7]. Executive functioning tests with ecological validity are often associated with instrumental activities of daily living (I-ADL) [7,8] and reflect the functional and predictive relationship between neurocognitive assessment performance and real-world behavior [9].

Despite the importance of neuropsychological assessments for predicting functional outcomes across personal, educational/occupational, and community contexts, few studies have systematically explored the ecological validity of different assessment tools or investigated the relationship between cognition and activities of daily living (ADL) [7]. In the case of children with ADHD, efforts have been made to conduct ecologically valid tests, primarily focusing on assessing sustained and selective attention [10] and effectively capturing the concept of ecological validity.

Recent advancements in virtual reality (VR) technology have provided promising avenues for creating immersive digital environments that simulate real-life scenarios [5]. VR environments use software to offer visual, auditory, and tactile stimuli, enabling individuals to interact within these simulated settings. The use of these enhances ecological validity by situating responses within specific contexts, involving meaningful tasks that replicate real-life activities and providing more precise information than self-reported measures that lack contextual specificity [6,7]. VR’s ability to standardize tests while manipulating environmental variables allows for a systematic investigation of their impact on cognitive performance, thereby enhancing our understanding of how these factors influence behavior [11]. Furthermore, VR’s immersive nature promotes participant engagement and naturalistic behavior, potentially improving assessment accuracy [12].

The proposed SmartAction-VR task aims to address these needs by assessing children’s and adolescents’ executive functioning through ecologically valid real-life tasks. The study hypothesizes that children and adolescents with ADHD exhibit lower performance and higher error rates in commission, omission, and rule-breaking behaviors than their neurotypical peers [7].

## 2. Materials and Methods

### 2.1. Design

A cross-sectional study [13] was conducted between November 2021 and December 2022 at the Faculty of Health Sciences of the University of Granada, Spain. 

### 2.2. Participants

Participants were selected using non-probability convenience sampling and divided into two groups: the ADHD group and the neurotypical group. To be included, they had to be between 9 and 17 years old. For the ADHD group, the inclusion criteria, in addition to age, included a clinical diagnosis of ADHD according to the ICD-10 (Attention Deficit Hyperactivity Disorder, F90.0). Participants with neurological disorders, such as photosensitive epilepsy and cerebral palsy, other motor disorders that impair upper limb mobility, balance, and gait, severe mental illnesses, or moderate to severe autism spectrum disorder, and those with an IQ < 80, were excluded. Participants in the ADHD group were diagnosed and recruited by pediatricians from local and primary care centers. The neurotypical group was recruited into primary, secondary, and high school settings through the Orientation Department.

To determine the socioeconomic status (SES) of the participants’ families, the highest level among the guardians was used. The SES was mainly derived from the occupation of guardians, following the classification of the Group on Social Determinants of the Spanish Society of Epidemiology from the National Classification of Occupations of 2011 [14], and was grouped into three levels: Level I (higher): directors, managers, and professionals with university degrees; Level II: intermediate occupations (employees, administrative and professional staff who support public administration and other services, and those who are self-employed); Level III (lower): manual workers.

### 2.3. Instruments

For this study, questionnaires on sociodemographic information (age, sex, educational level, and SES) and functional data (Waisman Activities of Daily Living Scale and Assessment of Sensory Processing and Executive Functioning in Childhood) were completed by the guardians. Additionally, a self-report (Pediatric Simulator Disease Questionnaire) was completed by the participants. Finally, cognitive tests (Digit Span Test, Stroop Test, Cognitive Flexibility Subtest, Trail Making Test, and Zoo Map) and the virtual task SmartAction-VR were administered to the participants by the evaluators. In addition, information was recorded on the primary diagnosis of the participants in the ADHD group (according to the International Classification of Diseases, ICD-10) [15] and whether or not they were receiving pharmacological treatment.

#### 2.3.1. Waisman Activities of Daily Living Scale (W-ADL) [16]

This questionnaire was completed by caregivers to report on the performance of their children and adolescents’ ADLs. It consists of 17 items and assesses both basic and instrumental ADL and the ability to perform tasks. Scoring of each item is on a scale of 0–2, as follows: 0 = does not do at all; 1 = does with help; 2 = independent or does on their own; higher scores indicate better performance. W-ADL has good internal consistency in different clinical populations, with a Cronbach’s alpha value between 0.77 and 0.94 [16].

#### 2.3.2. EPYFEI (Assessment of Sensory Processing and Executive Functioning in Childhood) [17]

This questionnaire was designed to assess sensory processing and executive functioning in children aged ≥ 3 years. The tool aims to capture how children process sensory information and how this relates to their executive skills. EPYFEI consists of 34 items that are grouped into five factors or dimensions: (1) executive attention; (2) sensory processing (particularly of tactile, proprioceptive, and vestibular information); (3) behavioral and emotional self-regulation; (4) problem-solving in ADLs; and (5) inhibitory control. In all dimensions of the EPYFEI, higher scores correspond to greater difficulty in the measured dimension, except for problem-solving, where higher scores reflect greater problem-solving ability. This distinction is considered when calculating the EPYFEI total score. For the EPYFEI total score, higher values indicate greater difficulties, with a cutoff point of ≥46.5 allowing for a differentiation between children and adolescents with and without difficulties in sensory processing and executive functioning in daily life. Psychometric results confirmed its internal consistency, test–retest reliability, construct validity, and discriminatory validity. Cronbach’s alpha for this questionnaire ranged from 0.70 to 0.95, and the test–retest reliability (ICC) was >0.70. Moreover, the EPYFEI had a high positive correlation with Short Sensory Profile-2, the Behavior Assessment System for Children (BASC), and the Childhood Executive Functioning Inventory (CHEXI) [17].

#### 2.3.3. Pediatric Simulator Disease Questionnaire (Peds-SSQ) [18]

This self-reporting tool was used to assess participants’ experiences when using VR. It consists of 13 items related to potential symptoms that might appear after using SmartAction-VR and is divided into four categories: eye strain (4 items), head and neck discomfort (2 items), fatigue or sleepiness (2 items), and dizziness or nausea (5 items). Participants indicate the severity of each symptom on a numerical scale from 0 to 6, where 0 means “no complaints” and 6 means “a lot of complaints”. Higher scores indicate lower tolerability to the VR experience [18].

#### 2.3.4. Digit Span Test [19]

This test measures attentional span and the ability to retain and manipulate information in working memory. The participant is presented with a sequence of digits in a specific order and is asked to repeat them both forward and backward. The higher the score, the better the performance. The test demonstrated very good reliability (Cronbach’s alpha = 0.83) [19].

#### 2.3.5. Stroop Test [20] 

This neuropsychological test assesses a person’s ability to process information and filter out irrelevant information in conflict situations. It is commonly used to determine whether children experience difficulties with selective attention and inhibitory control. Children taking longer to respond or making more errors indicates a reduced ability to inhibit automatic responses. The test–retest reliability of the Stroop test for children was robust, with correlation coefficients being generally > 0.80. The Stroop test correlates well with other measures of executive function and attention, demonstrating good convergent validity. Negative scores in the Stroop test (<0) indicate that the person has difficulty in inhibiting the interference caused by conflicting stimuli [20].

#### 2.3.6. Cognitive Flexibility Subtest of the NEPSY-II Neuropsychological Assessment Battery [21]

This test assesses a child’s ability to efficiently and flexibly switch from one task to another, a process known as “cognitive set shifting”. A specific subtest, “Auditory Attention and Cognitive Flexibility”, was used to evaluate the ability to quickly change tasks and inhibit incorrect responses. It consists of two parts: (1) the Auditory Attention Subtest, which assesses a child’s ability to process auditory information, discriminate between sounds, and pay attention to specific auditory stimuli, and (2) the Cognitive Flexibility Subtest, which evaluates a child’s ability to shift their thinking and adapt to new situations. The higher the number of errors, the lower the performance [21].

#### 2.3.7. Trail Making Test (TMT) [21,22]

This neuropsychological assessment test is used to evaluate cognitive function, visual attention, planning ability, and processing speed. Errors in the sequence of connections are also observed, which may indicate difficulties with visual attention and following instructions [21]. The internal consistency of the TMT was acceptable (Cronbach’s alpha above 0.70), and adequate test–retest reliability was found for Part A and Part B (r = 0.78 and 0.67, respectively). A higher number of errors indicates poorer performance, and longer task completion times reflect lower processing speed [21,22].

#### 2.3.8. Zoo Map Test [23] 

This is a subtest of the Behavioral Assessment of Dysexecutive Syndrome (BADS), a standardized battery of tests designed to assess EFs. The Zoo Map test specifically measures a subject’s ability to plan, organize, and solve problems through a visual–spatial task. It evaluates the ability to generate and maintain a plan, update it with new information, inhibit irrelevant information and responses, switch between tasks, and monitor performance [23]. This subtest demonstrated high test–retest reliability as well as inter-rater reliability, with correlation coefficients ranging from 0.88 to 1.00 [20,24]. The greater the number of errors, the lower the planning skills [23].

#### 2.3.9. SmartAction-VR

SmartAction-VR is a VR tool designed using the Unity 3D game engine (Unity Technologies, San Francisco, CA, USA) [25] to be implemented with Oculus Quest 2. The immersive task is based on the paradigm of the multiple errand test (MET), adapted to the cognitive instrumental activities of children and adolescents between 9 and 17 years, considering the demands of EFs. SmartAction-VR includes five scenarios: (1) Hall: the start and end point of the tasks from where the rest of the scenarios are accessed; (2) Bedroom: a scenario in which participants must fill a backpack and store the required school supplies; (3) Kitchen: a place where participants must prepare a sandwich with specific foods; (4) Street: a scenario that recreates a city with traffic, and participants must navigate and comply with pedestrian regulations to reach the stationery store; (5) Stationery store: a place where participants have to buy the books and school supplies they need, making an exact payment. All scenarios included distractors that were not part of any of the proposed tasks. The virtual task was performed in a 4 × 4 m laboratory. The time taken to perform SmartAction-VR ranged from 11 to 20 min, while the completion of the standardized test batteries required a run time of 20 to 30 min. The instructions for this task are presented in Table 1. A short video recording of SmartAction-VR can be accessed at the following link: https://www.youtube.com/watch?v=MbPfto8XZGM (accessed on 9 August 2024).

Each participant’s SmartAction-VR score was determined by tracking errors during virtual tasks, including the time taken to complete the task, the quality of performance, and types of errors made. Errors were classified according to a scheme previously used by other authors for naturalistic tasks [26,27]: omission, commission, estimation, not following rules, asking for help, questions, or comments. Omission errors refer to missing or incomplete actions needed to achieve a goal, such as forgetting school supplies when buying them or packing a backpack. Commission errors include perseverations, which involve the unnecessary repetition of actions; errors involving the inclusion of unnecessary or new actions not required by the task; and substitution errors, where an action or object is replaced by an unnecessary or incorrect one. Finally, three main variables were assessed: execution time (shorter is better), total errors (fewer is better), and accuracy, calculated as the number of successful actions divided by the total number of errors (higher is better).

### 2.4. Procedure

Evaluations were conducted by two psychologists and four occupational therapists. Participants in the ADHD group who were on medication were required to refrain from taking methylphenidate for at least 24 h before the assessment. The evaluations were conducted with the examiners blinded, i.e., unaware of each participant’s group assignment (ADHD or neurotypical). Each evaluation was performed in a single session divided into two parts and performed in a counterbalanced manner: some participants began with the cognitive tests and then completed SmartAction-VR, while others started with this task and completed the cognitive tests after.

Before beginning the SmartAction-VR task, the participant completed Peds-SSQ. Subsequently, the evaluator read the SmartAction-VR instructions (Table 1) aloud and asked the participant to explain the task to ensure understanding. After confirming comprehension, the participant was positioned in the center of the room and instructed to start the task. All actions, comments, questions, and errors were documented in a report. Upon completion of the task, Peds-SSQ was administered again to detect any new or altered symptoms related to VR use. The performance during the SmartAction-VR task was recorded in video format for subsequent error analysis. Throughout the assessments, the guardians provided clinical and sociodemographic data and completed the W-ADL and EPYFEI questionnaires.

### 2.5. Ethical Issues

Approval was obtained from the Ethics Committee of the local hospital (code: 22/07/2019/PEIBA/Report 7/19). Before the study, a meeting was held with the guardians of each participant to explain the project and its objectives and obtain their informed consent. All guardians provided written informed consent before the study began. Participant data were pseudonymized before the analysis.

### 2.6. Statistical Analysis

The initial statistical analyses addressed the following question: what is the distribution of the various variables included in the study? To this end, a descriptive analysis was performed to examine the distribution of variables for comparison purposes. For categorical variables (sex, ethnicity, educational level, and socioeconomic status), frequency and percentage were calculated, and differences in distribution between the comparison groups (ADHD vs. neurotypical) were analyzed using the Chi-square test or Fisher’s exact test when the expected values in any contingency table cell were less than five [28]. 

The Kolmogorov–Smirnov test with a Lilliefors correction was used to assess the normality of distributions for age, functional data (W-ADL and EPYFEI scores), Peds-SSQ scores, cognitive data (Digit Span Test, Stroop test, Cognitive Flexibility Subtest, TMT, and Zoo Map scores), and SmartAction-VR scores. Because these variables exhibited non-normal distributions (*p*-value of the Kolmogorov–Smirnov test < 0.05), the median and interquartile range (IQR) were computed. The Mann–Whitney U test was conducted to determine whether the ADHD group had lower performance and a higher number of errors in the SmartAction-VR task than the neurotypical group. Additionally, the Mann–Whitney U test was used to investigate if there were differences between the groups in the potential cybersickness effects by comparing the total and the four dimensions’ Peds-SSQ pre- and post-use scores for SmartAction-VR. Furthermore, the Wilcoxon signed-rank test was used to evaluate intragroup changes in the Peds-SSQ total scores and across its four dimensions before and after the task was performed. To address the question of whether it is possible to assess executive functioning in daily life with ecological validity through SmartAction-VR, the association between two quantitative variables was analyzed by determining the Spearman correlation coefficient between the results obtained in SmartAction-VR, the cognitive tests (Digit Span Test, Stroop Test, Cognitive Flexibility Subtest of the NEPSY-II, TMT A, TMT-B, and Zoo Map), and the results on functioning in daily life (W-ADL and EPYFEI). The significance level for all tests was set at *p* < 0.05. All statistical analyses were performed using IBM Statistical Package for Social Sciences Software (IBM Corp. Published in 2012. IBM SPSS Statistics for Windows, version 28.0 (Armonk, NY, USA). 

## 3. Results

The final sample consisted of 76 children and adolescents with a median age (IQR) of 13 [11,12,13,14] years and an age range of 9 to 17 years. Of these participants, 60.50% (*n* = 46) were male. This sample was divided into two groups: the ADHD group (*n* = 40) and the neurotypical group (*n* = 36). There were no statistically significant differences between the two groups in terms of ethnicity, educational level, or SES (Table 2). In the ADHD group, 63.4% (*n* = 26) were on methylphenidate as their usual pharmacological treatment (Table 2).

The results showed that the ADHD group took longer to complete both the sustained (TMT-A: U = 279, *p* < 0.001) and alternating (TMT-B: U = 175.5, *p* < 0.001) tests and made more errors (TMT-A: U = 576.5, *p* = 0.036; TMT-B: U = 335, *p* < 0.001). Scores on the Digit Span tests were higher for the neurotypical group (forward: U = 419, *p* = 0.001; backward: U = 491, *p* = 0.014). Additionally, statistically significant differences were found in interference inhibition (Stroop: U = 502.5, *p* = 0.034). The findings also showed that the ADHD group made more planning errors (Zoo Map errors: U = 278, *p* = 0.001) and had a longer execution time (Zoo Map execution time: U = 344.5, *p* = 0.001). Furthermore, the ADHD group exhibited more errors and difficulties in the flexibility subtests of NEPSY-II (U = 435, *p* = 0.006).

On the other hand, the ADHD group had a lower score on the W-ADL Scale [16] (U = 520, *p* = 0.037), indicating a greater need for support to perform ADL independently. Likewise, regarding EPYFEI [17], this group exhibited more difficulties in executive functioning and sensory processing in daily life, with the following results: executive attention, U = 87, *p* < 0.001; sensory processing, U = 431, *p* < 0.001; behavioral and emotional self-regulation, U = 440, *p* = 0.004; problem solving, U = 304.5, *p* < 0.001; inhibitory control, U = 248, *p* < 0.001; and EPYFEI total score, U = 159, *p* < 0.001 (Table 3).

All participants were able to perform the task without pauses and did not spontaneously report any symptoms of cybersickness during or after the task. Regarding Peds-SSQ, no significant differences were found in the scores of any dimensions or the total score between the two groups, either before or after performing SmartAction-VR. However, statistically significant intragroup differences were observed after performing it. In the ADHD group, a significant increase was observed in the fatigue score (Z = −1.992, *p* = 0.046), corresponding to an increase in the feeling of fatigue. In the neurotypical group, significant increases were observed in the neck discomfort (Z = −2.555, *p* = 0.011) and dizziness (Z = −2.315, *p* = 0.021) scores, suggesting an increase in these sensations.

In addition, the results of SmartAction-VR revealed significant differences between the comparison groups across various measures (Table 4). The ADHD group exhibited more total errors (U = 292, *p* = 0.001). Furthermore, it demonstrated lower accuracy than the neurotypical group (U = 406, *p* = 0.010). In terms of error types, the findings indicated that the ADHD group made more commissions (U = 417, *p* = 0.003), more new actions (U = 470, *p* = 0.014), and more forgetting actions (U = 406, *p* = 0.010), but fewer perseverations (U = 540.5, *p* = 0.029) than the neurotypical group (Table 4).

On the other hand, after performing SmartAction-VR, the findings revealed that individuals with higher accuracy had lower scores in difficulties related to the executive attention factor (r = −0.376, *p* = 0.001), higher scores in the problem solving factor (r = 0.396, *p* < 0.001), and a shorter execution time on the Zoo Map (r = −0.316, *p* = 0.007). Additionally, those with more total errors performed worse on the Flexibility Subtest (r = 0.533, *p* < 0.001), experienced greater difficulties with the executive attention (r = 0.425, *p* < 0.001), sensory processing (r = 0.400, *p* < 0.001), behavioral and emotional self-regulation (r = 0.380, *p* < 0.002), and inhibitory control factors (r = 0.398, *p* < 0.001), and had poorer performance in sustained attention in TMT A time (r = 0.347, *p* < 0.004) and in alternating attention in terms of TMT B errors and time (r = 0.401, *p* < 0.001 and r = 0.440, *p* < 0.001, respectively). Those who made more omission errors had lower scores in the behavioral and emotional self-regulation factor (r = −0.396, *p* < 0.001) and the problem-solving factor (r = −0.345, *p* = 0.005). Similarly, higher scores for forgetting actions were associated with greater scores in sustained attention in terms of TMT A time (r = 0.310, *p* < 0.009), and lower scores in alternating attention in terms of TMT B time (r = −0.313, *p* = 0.008), the problem-solving factor (r = −0.319, *p* = 0.010), and the behavioral and emotional self-regulation factor (r = −0.396, *p* < 0.001) (Table 5). 

In contrast, individuals who made more commission errors exhibited worse performance in sustained attention in terms of TMT A time (r = 0.327, *p* < 0.004) and in alternating attention in terms of TMT B time and errors (r = 0.407, *p* < 0.001 and r = 0.446, *p* < 0.001, respectively), and had more difficulties in the executive attention (r = 0.416, *p* < 0.001), sensory processing (r = 0.368 *p* < 0.001), behavioral and emotional self-regulation (r = 0.365 *p* < 0.001) and inhibitory control factors (r = 0.457, *p* < 0.001) (Table 5). 

Additionally, those who made more substitution errors performed worse on the Flexibility Subtest (r = 0.366, *p* = 0.001) and had more difficulties in terms of the inhibitory control factor (r = 0.308, *p* < 0.007). Individuals with more errors in new actions showed poorer performance in alternating attention in terms of TMT B errors and time (r = 0.350, *p* = 0.002 and r= 0.413, *p* < 0.001, respectively) and in the Flexibility Subtest (r = 0.465, *p* < 0.001), and had more difficulties with the executive attention (r = 0.345, *p* = 0.002), sensory processing (r = 0.319, *p* = 0.005), behavioral and emotional self-regulation (r = 0.319, *p* = 0.005) and inhibitory control factors (r = 0.392, *p* < 0.001). Furthermore, participants with more errors related to not following the rules (broke rules) experienced greater difficulties with the problem-solving (r = −0.382, *p* < 0.001) and inhibitory control factors (r = 0.317, *p* = 0.006). Those who required more help, performed better in the Flexibility Subtest (r = −0.338, *p* = 0.003). Finally, higher accuracy was also associated with higher scores in W-ADL (r = 289, *p* < 0.015) and lower scores in omission errors (r = − 0.336, *p* < 0.007), forgetting actions (r = −0.281, *p* < 0.024), and commission errors (r = −0.261, *p* < 0.024) (Table 5).

## 4. Discussion

This study assessed cognitive performance in children and adolescents aged 9–17 years using SmartAction-VR, a new tool for evaluating EFs in daily life. This VR system was designed for ecological assessments of EFs, replicating meaningful everyday situations for children and teens, including schoolwork and social/household activities. The preliminary results suggest that SmartAction-VR could be a useful tool for assessing EF performance in individuals with ADHD, distinguishing them from the neurotypical population. Additionally, these results align with recent studies showing the positive acceptability of digital and technological assessment tools over traditional paper-based tests [29]. Unlike other available tools, SmartAction-VR allows activities to be performed in different scenarios, following the multiple errands paradigm, and gives individuals freedom of movement. They can decide whether to move faster or slower, perform activities in a specific sequence, or alter them. Another important characteristic is that activities are initiated by the participant, similar to what happens in ADL [8,30]. This type of task identifies specific problems through realistic scenarios, therefore helping clinicians design intervention strategies that can be integrated into the child’s daily routines, and thereby improving the person’s overall functioning [31].

In SmartAction-VR, the ADHD group showed longer execution times, more total errors (including commissions and omissions), greater difficulty following rules, and higher rates of substitutions, introducing new actions, and forgetting actions compared to the neurotypical group. These findings support the existence of cognitive differences in EF in daily life between individuals with ADHD and their neurotypical peers [32]. Similar results have been partially reported by other researchers using a comparable task conducted by children with ADHD, in whom the number of irrelevant actions was higher compared to those that in those with neurotypical development [33]. 

VR tasks to assess ADHD have recently emerged; however, many of them focus on sustained attention tasks in unrealistic settings [34]. VR assessment instruments developed so far exhibit limited ecological validity, as their interfaces present scenarios and activities that bear little or no relation to actual daily life activities [35]. They also fail to incorporate natural types of movement (such as navigating through different scenarios and manipulating various materials) and real-life distractors [35], except for a new task developed to assess prospective memory [33,36].

VR tools have the potential to create immersive environments where individuals perform tasks that reflect everyday activities, providing more accurate data on their functional cognition [7,36,37]. SmartAction-VR aims to address this gap by offering a new tool to evaluate EF with ecological validity. It considers both task type and meaningfulness and enhances interaction within the task itself, thereby increasing the sense of presence. This allows individuals to move naturally and perform I-ADL as they would in their own environments [7,30]. 

The performance of ADL in the ADHD group was lower than that of neurotypical participants, which is consistent with findings from other studies [38,39]. These results are consistent with those observed using SmartAction-VR. Furthermore, the ADHD group exceeded the cut-off score of 46.5 for the EPYFEI [17], indicating that individuals with ADHD had greater deficits in EF and sensory processing compared to their neurotypical peers in daily life. These findings are also consistent with the results of other studies [39,40,41,42].

Regarding the correlations found between the results of the neuropsychological tests administered and the performance of SmartAction-VR (Table 5), there was a significant negative correlation between accuracy and the total errors, omissions, and commissions. these findings are also supported by other authors [34,43]. Participants with ADHD were more likely to forget actions, which is consistent with deficits in prospective memory observed in task performance [44]. 

This study presents preliminary findings with the developed SmartAction-VR task. However, this study has several limitations. First, the sample was selected using non-probability convenience sampling, which may have limited the extrapolation of results. However, its usefulness in exploratory studies, such as ours, was demonstrated [45]. Another limitation was the small sample size, due to the duration of the project (just over a year) and the availability of families to take their children for evaluation within this period. This could have reduced the statistical power of the study, potentially lowering the probability of detecting a true effect if one exists and reducing the likelihood that a statistically significant result reflects a real effect [46]. Consequently, this could limit the generalizability of the results, too. 

To address these limitations, future studies could benefit from an extended recruitment period, diversified recruitment channels, stratified random sampling, and the calculation of statistical power to determine the sample size needed to detect significant findings. As future lines of research, it would also be interesting to study functional cognition in other children with neurodevelopmental disorders using SmartAction-VR, as well as in other age groups, as explored in similar research [36], and to study the psychometric properties of SmartAction-RV. Furthermore, it could be of interest to design studies aimed at evaluating the diagnostic capability of VR tasks similar to SmartAction-VR, incorporating principal component analysis (PCA) and specificity tests.

## 5. Conclusions

SmartAction-VR is a new tool developed to assess executive functioning in daily life. This study has shown that the types of cognitive errors observed in SmartAction-VR among children and adolescents with ADHD are similar to those detected through traditional cognitive tests. Specifically, participants with ADHD exhibited a greater number of total errors, including commissions and omissions, than the neurotypical group. Additionally, the number of errors related to forgetting actions and omissions in this virtual task was associated with the level of independence in ADL.

## Figures and Tables

**Table 1:** SmartAction-VR instructions.

Next, you will see that you are in the corridor of an apartment building, where there are three doors, one corresponds to the kitchen, another to your room and the third is the door to the street. On the street, there are various shops, including a stationery store. Your mission is to complete the following three tasks:

Prepare a backpack for the school/high school Buy the necessary materials that you do not have in your room Prepare a ham and cheese sandwich and leave it on the counter

In the backpack you must include the following items: - Math book - Language book - English book - Geography and History Book - English notebook - Red marker - Blue pen - Book titled “The Dream of Berlin”

You will find some of these items in your room, but others may be missing and need to be purchased at a stationery store.

The task involves a series of rules that you must follow: - Be cautious with traffic lights and cars. Follow traffic regulations and do not cross the street if the pedestrian light is not green. - To walk on the street, press the upper button on the controller held in your left hand, following the red laser line. - To return home, follow the red laser line and approach or stand near a green triangle that you will see on the street. - To pay at the bookstore, press the upper button on the controller held in your left hand. Ensure to leave the exact amount of money on the counter.

Once you have paid, the bookseller will pack your purchase in a bag. You only should take the bag; you do not have to put the items in the backpack.

The three tasks can be completed in any order you prefer. You have 20 min to finish them. At the end of the 20 min, return to the corridor of your apartment, where you will see a television on. When you have finished, let us know by saying: I have finished!”

**Table 2:** Characteristics of the sample (*n* = 76).

	Neurotypical (*n* = 36) *n* (%)	ADHD (*n* = 40) *n* (%)	Chi-Square/U Mann–Whitney/Fisher Exact Test	*p*-Value
**Gender**			3.172 ^a^	0.075 ^a^
Girls	18 (50)	12 (40)		
Boys	18 (50)	28 (60)		
**Age (** **Median; IQR)**	13 (12–14)	12.50 (10–14)	−1.391 ^b^	0.169 ^b^
**Ethnicity**			1.199 ^c^	1 ^c^
Caucasian	35 (97.20)	37 (92.5)		
African	1 (2.80)	2 (5)		
Hispanic American	0 (0)	1 (2.5)		
**Educational level**			0.430^c^	0.902 ^c^
Primary	11 (30.50)	14 (35)		
Secondary	24 (66.7)	25 (62.5)		
High School	1 (2.8)	1 (2.5)		
**SES**			4.045 ^c^	>0.107 ^c^
Level I	18 (50)	12 (30)		
Level II	18 (50)	26 (65)		
Level III	0 (0)	2 (5)		
**Pharmacological treatment**				
(methylphenidate)				
Yes	-	26 (63.4)		
No	-	14 (36.6)		

ADHD: attention deficit hyperactivity disorder; IQR: interquartile range (25th–75th); ^a^ Pearson ‘s Chi-square test; ^b^ U Mann–Whitney test; ^c^ Fisher’s exact test; SES: socioeconomical status; Level I: directors, managers, and professionals with university degrees; Level II: intermediate occupations (employees, administrative and professional staff who support public administration and other services; and those who are self-employed); Level III: manual workers.

**Table 3:** Performance on measures of executive functioning.

	Neurotypical (*n* = 36)		ADHD (*n* = 40)			
	Median	(IQR)	Median	(IQR)	U	*p*
TMT-A Time	29	(25–35)	44.35	(34–64)	279	<0.001
TMT-A Errors	0	(0–0)	0	(0–1)	576.5	0.036
TMT-B Time	70	(59–70)	122	(85.75–167.50)	175.5	<0.001
TMT-B Errors	0	(0–0)	2	(0–5)	335	<0.001
Digit Span Forward	5	(5–6)	4	(4–5)	419	0.001
Digit Span Backward	4.5	(4–5)	4	(3–5)	491	0.014
Stroop	8	(5–8)	6	(–1–9)	502.5	0.034
Zoo Map Planning Time	61.50	(46.22–129.75)	64	(39–115)	670	0.603
Zoo Map Execution Time	33	(23.02–39.75)	61	(38.75–82.25)	344.5	0.001
Zoo Map Errors	0	(0–0)	2	(0.25–2)	278	0.001
Auditory Attention Errors	0.50	(0–1)	1	(0–3)	498	0.051
Flexibility Errors	1	(0–2)	2	(0.75–6.25)	435	0.006
W-ADL	28.50	(23–31)	24.50	(20.50–29.75)	520	0.037
Executive Attention	4.5	(1–6.75)	24.50	(15.25–33)	87.5	<0.001
Sensory Processing	0	(0–0)	1	(0–2)	431	<0.001
Behavioral and Emotional Self-Regulation	4.5	(2–8.75)	9	(4.25–14.50)	440	0.004
Problem Solving	19	(17–22)	14	(10.25–17)	304.5	<0.001
Inhibitory Control	1	(0–3)	12	(3.25–15.75)	248	<0.001
EPYFEY Total Score	30.50	(26.5–36)	57.5	(48.50–75)	159	<0.001

IQR: interquartile range (25th–75th); U: value of the U statistic obtained from the Mann–Whitney test.

**Table 4:** Performance in SmartAction-VR.

	Neurotypical (*n* = 36)		ADHD (*n* = 40)			
	Median	(IQR)	Median	(IQR)	U	*p*
Execution Time	15	(13.25–19.75)	17.5	(15–20.75)	556.5	0.087
Total Errors	6	(4.25–9.75)	11	(7–21)	292	0.001
Accuracy	0.86	(0.83–0.93)	0.83	(0.75–88)	406	0.010
Commission	4	(2–7)	8	(3–17.75)	417	0.003
Broke Rules	0	(0–1)	1	(0–1)	544	0.063
Perseverations	1	(0–1)	0.0	(0–0)	540.5	0.029
Substitution	0.50	(0–2)	1	(0–2)	575	0.109
New Actions	2	(0–5)	6	(1–17.5)	470	0.014
Spatial Estimation	10	(4–15)	14.5	(6.25–22)	547	0.104
Omission	6	(3–7)	7	(5–8.75)	475	0.078
Forgetting Actions	4	(2–5)	7	(3.5–7.5)	406	0.010
Forgetting Materials	1	(1–2)	1	(0–2)	497	0.364
Help	3.5	(2–6)	1	(0.25–2.75)	640.5	0.403

IQR: Interquartile Range (25th–75th); U: Value of the U statistic obtained from the Mann–Whitney test.

**Table 5:** Relationship between performance in SmartAction-VR and cognitive tests.

	Execution Time	Total Error	Accuracy	Comission	Broke Rules	Perseveration	Substitution	New Action	Omission	Forgetting Action	Forgetting Materials	Help
TMT A (Time)	0.270 *	0.347 **	−0.310 **	0.327 **	0.180	−0.165	0.192	0.261 *	0.163	0.310 **	−0.177	−0.168
TMT A (Errors)	0.111	0.210	−0.046	0.226	0.033	−0.133	0.183	0.265 *	−0.071	0.046	−0.125	−0.050
TMT B (Time)	0.363 **	0.440 **	−0.313 **	0.407 **	0.221	−0.159	0.297 **	0.350 **	0.168	−0.313 **	−0.170	−0.090
TMT B (Errors)	0.334 **	0.401 **	−0.159	0.446 **	0.098	−0.074	0.296 **	0.413 **	0.008	0.159	−0.208	−0.128
Direct Digits	−0.399 **	−0.215	0.154	−0.256 *	−0.118	0.132	−0.033	−0.235 *	−0.082	−0.154	0.034	0.031
Backward Digits	−0.365 **	−0.297 *	0.304 **	−0.238 *	−0.106	0.076	−0.159	−0.201	−0.223	−0.304 **	0.027	0.264 *
Stroop	−0.117	−0.184	0.102	−0.106	−0.257 *	−0.119	−0.066	−0.066	−0.086	−0.102	0	0.125
Planning Time Zoo Map	0.062	−0.076	0.097	−0.109	−0.167	−0.182	0.056	−0.052	−0.090	−0.097	0.005	−0.041
Execution Time Zoo Map	0.141	0.092	−0.316 **	0.115	0.053	−0.169	0.085	0.055	0.279 *	0.316 **	0.109	−0.009
Errors Zoo Map	0.097	0.188	−0.235 *	0.174	0.226	−0.185	0.114	0.120	0.150	0.235 *	−0.002	−0.113
Auditory Attention NEPSY-II	0.149	0.280 *	−0.005	0.259 *	−0.088	−0.156	0.225	0.280 *	−0.083	0.005	−0.254	−0.207
Flexibility NEPSY-II	0.302 **	0.533 **	−0.242 *	0.505 **	0.201	0.035	0.366 **	0.465 **	0.093	0.242 *	−0.281 *	−0.338 **
W-ADL	0.188	−0.232	0.289 *	−0.261 *	−0.194	0.036	−0.120	−0.217	−0.336 *	−0.281 *	−0.094	−0.283 *
Executive Attention	0.160	0.425 **	−0.376 **	0.416 **	0.276 *	−0.225	0.212	0.345 **	0.172	0.172	−0.145	−0.168
Sensory Processing	0.367 **	0.400 **	−0.172	0.368 **	0.131	0.014	0.269 *	0.319 **	0.202	0.202	−0.129	−0.079
Behavioral and Emotional Self-Regulation	0.310 **	0.380 **	−0.202	0.365 **	0.175	0.134	0.208	0.319 **	−0.396 **	−0.396 **	0.052	−0.122
Problem-Solving	−0.035	−0.194	0.396 **	−0.225	−0.382 **	0.248 *	−0.129	−0.164	−0.345 **	−0.319 **	−0.222	−0.053
Inhibitory Control	0.219	0.398 **	−0.311 **	0.457 **	0.317 **	−0.072	0.308 **	0.392 **	0.303	0.303*	−0.193	−0.227 *
EPYFEI Total Score	0.254*	0.461 **	−0.303*	0.441 **	0.180	−0.063	0.275 *	0.372 **	0.376 **	0.376 **	−0.165	−0.077

* The correlation is significant at the level of 0.05 (bilateral); ** The correlation is significant at the level of 0.01 (bilateral).

## Data Availability

The data presented in this study are only available on request from the corresponding author due to ethical reasons.
